# The Predictive Value of Urinary Kidney Injury Molecular 1 for the Diagnosis of Contrast-Induced Acute Kidney Injury after Cardiac Catheterization: A Meta-Analysis

**DOI:** 10.1155/2020/4982987

**Published:** 2020-08-13

**Authors:** Qing Li, Yimin Huang, Weifeng Shang, Ying Zhang, Yanyan Liu, Gang Xu

**Affiliations:** ^1^Department of Nephrology, Tongji Hospital, Tongji Medical College, Huazhong University of Science and Technology, Wuhan 430030, Hubei Province, China; ^2^Department of Nephrology, Medical Intensive Care, Charité-Universitätsmedizin Berlin, Berlin, Germany; ^3^Cellular Neurosciences, Max Delbrück Center for Molecular Medicine, Berlin, Germany; ^4^Department of Nephrology and Rheumatology, Puai Hospital Affiliated with Tongji Medical College, Huazhong University of Science and Technology, Wuhan 430000, Hubei Province, China

## Abstract

**Background:**

Urinary kidney injury molecule 1 (uKIM-1) is a proximal tubular injury biomarker for predicting acute kidney injury (AKI); its prognostic value varies depending on the clinical and population characteristics. However, the predictive value of uKIM-1 for diagnosis of contrast-induced acute kidney injury (CI-AKI) remains unclear.

**Method:**

Medline, Embase, ClinicalTrials.gov, Cochrane Library database, and the China National Knowledge Infrastructure (CNKI) were used to identify relevant studies from their inception to November 31, 2019. Studies that met the inclusion criteria were included. Relevant data were extracted to obtain pooled sensitivity (SEN) and specificity (SPE), summary receiver operating characteristic curve (ROC), and area under the ROC (AUC or AUROC). A bivariate mixed-effects regression model was used for data analysis.

**Results:**

A total of 946 patients from 8 eligible studies were included. Across all the studies, the diagnostic odds ratio (DOR) for uKIM-1 level to predict CI-AKI was 19 (95% CI 10–39), with SEN and SPE of 0.84 and 0.78, respectively. The AUROC for uKIM-1 in predicting CI-AKI was 0.88 (95% CI 0.85–0.90). There was a substantial heterogeneity across the studies (*I*^2^ was 37.73% for the summary sensitivity and 69.31% for the summary specificity).

**Conclusion:**

Urinary KIM-1 has a high predictive value for diagnosis of CI-AKI in patients who have undergone cardiac catheterization.

## 1. Introduction

Contrast-induced acute kidney injury (CI-AKI) is a common and serious complication after cardiac catheterization (CC), percutaneous coronary intervention (PCI), and coronary angiography (CAG). It accounts for 12% of all hospital-acquired kidney failure and increases the length of hospitalization, a situation that is worsening with increasing numbers of patients with comorbidities, including those requiring cardiovascular interventional procedures [1]. Unfortunately, current preventive methods for CI-AKI are limited and often ineffective [2]. Thus, the early and effective diagnosis of CI-AKI is important. Currently, the concentration of serum creatinine (sCr) is the most commonly accepted clinical standard for diagnosis of CI-AKI and it has several limitations. For example, sCR is affected by many factors (e.g., age, sex, race, weight, and drugs) [3]. Moreover, it takes over 24 to 48 hours to diagnose CI-AKI using sCr, which makes it too late for intervention [4]. Thus, sCr is believed to be inadequate for the diagnosis of CI-AKI, and there is an urgent need for biomarkers that can detect CI-AKI sooner and more accurately.

Kidney injury molecule 1 (KIM-1) is a phosphatidylserine receptor that is expressed in epithelial cells. It enables epithelial cells to recognize and phagocytose dead cells that are present in the stressed kidney [5]. KIM-1 is undetectable in the urine of normal subjects [6]. However, following renal tubular injury, KIM-1 is upregulated and delivered into the extracellular space and the urine, making it possible to detect renal damage [6, 7]. Previous reports have demonstrated that KIM-1 is an excellent predictor of proximal tubular injury compared with sCr in rats [7]. Several human studies have also shown that urinary KIM-1 (uKIM-1) can be detected well before the increase of sCr, thus making it a sensitive and specific early biomarker of CI-AKI [8–10]. Recently, reviews have shown that uKIM-1 is a promising biomarker for the diagnosis of acute kidney injury (AKI) [1, 11]. However, the predicative value of uKIM-1 for CI-AKI after percutaneous coronary intervention (PCI) or coronary angiography (CAG) still remains to be established. This meta-analysis was conducted to determine the predictive ability of uKIM-1 for the diagnosis of CI-AKI following PCI or CAG based on research evidence currently available.

## 2. Methods

### 2.1. Search Strategy

This meta-analysis was performed according to the guideline of PRISMA statement [12]. Relevant articles were identified through a systematic search of PubMed, Medline, Embase, Cochrane Library, ClinicalTrials.gov, and the China National Knowledge Infrastructure (CNKI) databases from their inception to November 31, 2019, with no language restriction. The following search terms were used: (KIM1OR KIM-1 OR kidney injury molecule 1) AND (contrast OR radio contrast OR contrast media OR contrast medium [CM] OR radio-contrast media OR radio contrast medium OR contrast agent] AND (acute kidney injury OR AKI OR acute kidney failure OR renal failure) AND (coronary angiography OR percutaneous coronary interventions OR cardiac catheterization). References of relevant studies were searched manually to identify eligible studies. In addition, abstracts were included in this meta-analysis if they contained complete results sections. The search was performed by two investigators (QL and YH).

### 2.2. Study Selection

Studies were assessed and selected based on titles and abstracts by two independent reviewers; conflicts were resolved by a third reviewer (YL). The inclusion criteria were as follows:

1. uKIM-1 was used as a biomarker to diagnose CI-AKI after PCI or CAG.
2. The study should be observational.
3. uKIM-1 concentration values for true-positive, false-negative, false-positive, and true-negative were provided or could be calculated to predict CI-AKI.

The exclusion criteria were as follows:

1. Review articles or duplicate papers
2. Animal or in vitro based studies
3. Studies that did not provide the diagnostic accuracy of uKIM-1

### 2.3. Data Extraction and Study Quality Assessment

After a detailed full-text review of each study, data was extracted from the retained studies by two independent reviewers (QL and YH). Disagreements were resolved by discussion and assessed by a third reviewer (YL) until a consensus was reached. The following data were extracted from the included studies for analysis: first author, year of publication, original country, sample size, study design, incidence of CI-AKI, inclusion renal criteria, patient characteristics (age, sex, baseline serum creatinine, and the proportion of patients with hypertension and diabetes mellitus), definition of CI-AKI, and CM (contrast media) type. Information about biological material, measurement assay, and time of uKIM-1 management was also collected. In addition, sensitivity and specificity from the cutoff value of the uKIM-1 level predicting CI-AKI (obtained by receiver operating characteristic (ROC) analysis and the area under the curve (AUC) of ROC) were extracted from the studies.

The methodological quality of each included study was assessed by two independent reviewers (QL and YH) who used the Quality Assessment of Diagnostic Accuracy Studies-2 (QUADAS-2) tool [12].

### 2.4. Statistical Analysis

Statistical analyses were performed using STATA version 14.0 (Stata Corp, College Station, TX), notably with the “midas” commands [13]. An exact binomial rendition of the bivariate mixed-effects regression model that was developed by von Houwelingen et al. [14] for meta-analysis of treatment trials and modified for diagnostic test data using an approximate normal within study model was used [15]. The bivariate mixed-effects model fits a 2-level model, with independent binomial distributions for the true positives and true negatives conditional on the sensitivity and specificity in each study, and a bivariate normal model for the logit transformations of sensitivity and specificity between studies [13]. Based on this model, the pooled sensitivity (SEN), pooled specificity (SPE), positive likelihood ratio, negative likelihood ratio, and diagnostic odds ratio (DOR) with their 95% CIs were obtained. We also constructed hierarchical summary ROC curves to plot sensitivity versus specificity and calculated the AUC [16]. The degree of the heterogeneity, which indicates the variation of included studies, was assessed using the *I*^2^ statistic [15]. I^2^ describes the percentage of total variation across studies that is attributable to the heterogeneity rather than to chance. The value of *I*^2^ lies between 0% and 100%, a value of 0% indicates no observed heterogeneity, and values greater than 50% may be considered substantial heterogeneity [13]. In addition, we conducted a subgroup analysis according to the detection time, sample size, and presence of chronic kidney disease. Publication bias was evaluated using Deek's effective sample size funnel plot.

## 3. Results

### 3.1. Study Selection

**Figure 1:**
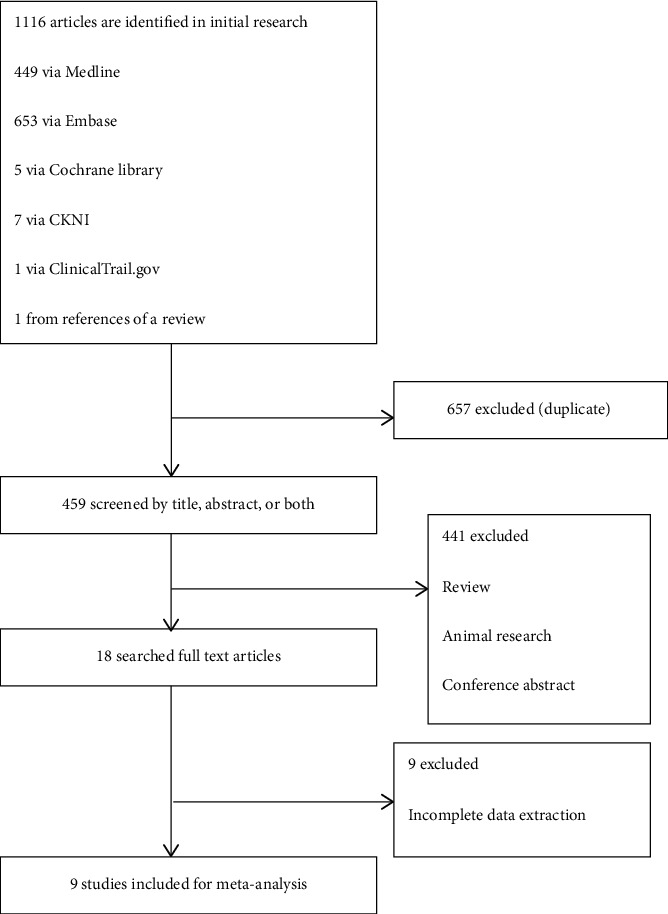
Flow diagram for the review process and outcomes of inclusion and exclusion.

The study selection process is shown in Figure 1. A total of 1116 publications from different databases were retrieved upon initial search. Of those, 657 articles were excluded due to duplication. The remaining studies were screened by title and/or abstract; 441 of them were removed because they were reviews, animal research, or conference abstracts. Of the remaining 18 studies, 9 were excluded due to missing essential data (e.g., SEN, SPE, and diagnostic criteria used). In addition, one article [17] was found during the manual review of the references of a review article [1] and included in this meta-analysis. In summary, 9 original studies [8–10, 17–22] were included in this meta-analysis.

### 3.2. Study Characteristics

All studies were single-center trials that had their findings published between 2013 and 2017. Of the included studies, 7 were published in English [8–10, 17–19, 22], and 2 were published in Chinese [20, 21]. The 9 studies included a total of 946 adult patients from 5 countries who underwent PCI or GAC, and 144 were diagnosed as CI-AKI. Among these studies, 8 included patients with normal sCr levels and 1 [10] included CKD patients. Patients treated with dialysis or who used renal toxic drugs were excluded from all studies. However, the definition of CI-AKI differed among the studies. Six of the studies used a traditional definition for CI-AKI (increase in SCr ≥ 0.5 mg/dL or ≥ 25% within 48–72 hours after contrast exposure) [4], while three studies did not [8, 17, 19]. The characteristics of all the individual studies are listed in Table 1.

### 3.3. Methodological Quality of Included Studies

The methodological quality assessment for the studies included in this meta-analysis is shown in Table S1. The overall quality of the eligible studies was not robust. Most studies showed low risk in the domain of patient selection, while three studies [8, 18, 20] that did not avoid a case-control design showed high risk. An unclear risk was shown in the domain of index test since most of the studies did not indicate if a threshold was used. An unclear risk was also shown in the domain of risk of bias because information about whether reference standard results were interpreted without knowledge of the results of the index test was not provided in most studies. There was high risk in the domain of flow and timing because two studies [10, 20] did not include all the patients in the analysis. All the studies were regarded to be of low risk for concerns regarding applicability.

### 3.4. Result of Individual Studies

The performance of uKIM-1 concentrations for predicting CI-AKI has been summarized in Table 2. Urinary KIM-1 levels were measured in all of the studies. Enzyme-linked immunosorbent assay (ELISA) was used in all of the studies to evaluate the level of uKIM-1. The time of the uKIM-1 test ranged from 6 to 24 hours after PCI or GAC. The cutoff value of the uKIM-1 level used to predict CI-AKI varied from 0.048 to 6.33 ng/ml, and one study even set the cutoff value as 3 times of the baseline uKIM-1. Moreover, the AUC of uKIM-1 for predicting CI-AKI ranged from 0.713 to 0.95.

### 3.5. Meta-Analysis

**Figure 2:**
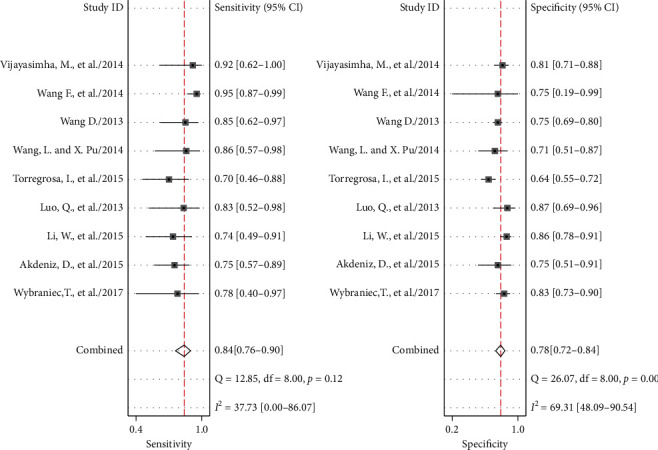
Forrest plots of the sensitivity and specificity of each individual study, summary sensitivity and specificity, and I^2^ statistic for heterogeneity.

**Figure 3:**
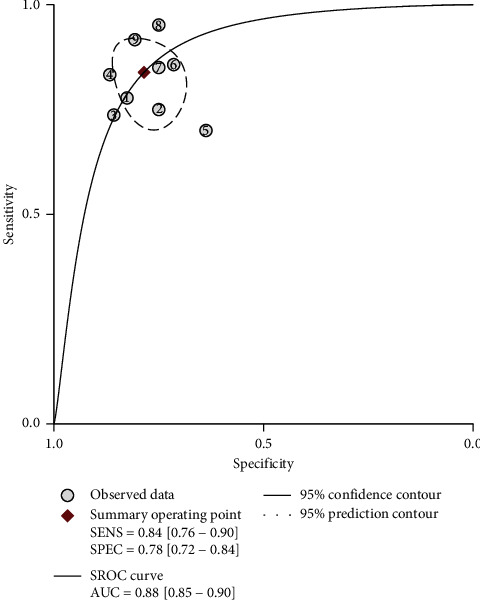
Summary receiver operating characteristic (SROC) graph with 95% confidence region and 95% prediction region for the diagnosis value of CI-AKI by KIM-1.

As shown in Figure 2, the pooled sensitivity and specificity of all studies was 0.84 (95% CI 0.76–0.90) and 0.78 (95% CI 0.71–0.84), respectively. *I*^2^ was 37.73 for the summary sensitivity and 69.31 for the summary specificity, suggesting a substantial heterogeneity in the sample of studies. The area under the receiver operating characteristic curve (AUROC) was 0.88 (95% CI 0.84–0.90). The SROC graph with the 95% confidence region and the 95% prediction region are shown in Figure 3.

A subgroup analysis was performed to explore potential sources of heterogeneity among studies. As shown in Table 3, the diagnostic accuracy of uKIM-1 was higher in test time of <24 hour group than test time at 24 hour group. However, the opposite findings were found in the subgroups with CKD and large sample sizes.

**Figure 4:**
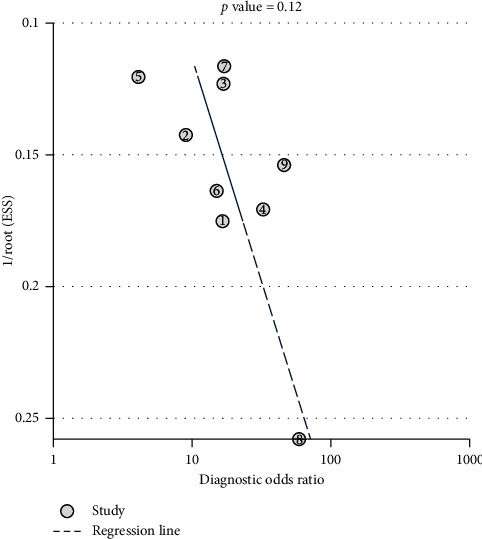
Deek's funnel plot asymmetry test.

The result of the Deek's funnel plot asymmetry test with a superimposed regression line is shown in Figure 4. The *P* value for the slope coefficient was 0.12, indicating nonsignificant asymmetry. This result indicates no potential publication bias among studies.

## 4. Discussion

In recent years, the gold standard for AKI diagnosis, the concentration of serum creatinine, has showed many limitations. Thus, several new biomarkers for early detection of AKI have been studied. Among them, the most promising are neutrophil gelatinase-associated lipocalin (NGAL) and kidney injury molecule 1 (KIM-1).

KIM-1 is a phosphatidylserine receptor and expressed in epithelial cells. It can recognize and phagocytose dead cells that are present in the stressed kidney [5]. In normal situations, KIM-1 is undetectable in urine. But when the proximal tubule suffers from ischemic or toxic injury, the ectodomain of KIM-1 is shed into the urine of humans [6, 7]. As a result, it is possible to use urinary KIM-1 (uKIM-1) to predict tubular injury and AKI.

A number of clinical studies have reported the excellent diagnostic value of uKIM-1 level for AKI caused by different reasons and in different populations [23–25]. The findings from a meta-analysis evaluating the predictive value of uKIM-1 in the setting of AKI, which included 2979 patients from 11 studies, suggest it is an efficacious diagnostic marker for AKI [11]. However, AKI caused by use of imaging contrast has not been systematically assessed to date. Thus, we performed a systematic and comprehensive review to investigate the diagnostic value of uKIM-1 for early detection of CI-AKI in patients who undergo PCI or CAG. To our knowledge, this is the first meta-analysis performed to evaluate the ability of uKIM-1 to predict CI-AKI.

Although we tried our best to search for all the eligible studies, there were only 9 that met our inclusion criteria and were included in the meta-analysis. Fortunately, these 9 studies showed a considerate homogeneity, indicating that the pooled results in this meta-analysis were reliable and stable.

In our study, SEN, SPE, and AUC were used for indicating the performance of uKIM-1 for CI-AKI prediction; all the studies showed that uKIM-1 was a good predictor. The AUC values were above 0.70 for all studies (in fact, two studies reported an AUC >0.90(18, 22)). Moreover, the pooled AUC (0.88, 95% CI 0.85–0.90) also indicates a considerable value in using uKIM-1 for the diagnosis of CI-AKI. Ho et al. constructed a meta-analysis of using uKIM-1 for diagnosis of AKI associated with cardiac surgery [26]. In this meta-analysis, the AUC of uKIM-1 for intraoperative and postoperative AKI were 0.68 (0.58–0.78) and 0.72 (0.69–0.84), respectively. There was also a meta-analysis carried out by Wang et al. that assessed the predictive value of NGAL for CI-AKI and showed an AUC of 0.93 [27]. In comparison with the aforementioned findings, we ascertained that uKIM-1 was much more effective for the diagnosis of CI-AKI than for cardiac surgery associated AKI but less powerful than NGAL for predicting CI-AKI.

Urinary KIM-1 was reported to be influenced by detection time. In a cohort of 103 adults undergoing cardiopulmonary bypass (CPB), the uKIM-1 levels increased by approximately 40% at 2 hours postoperatively and by more than 100% at the 24-hour time point [28]. In a nested case-control study consisting of cardiac catheterization patients, uKIM-1 increased 6 hours after contrast from the baseline and peaked at 24 hours, suggesting it may perform well in the relatively late time period (rather than at 6 hours) after AKI (19). We also found that 24-hour postcontrast exposure time was the best detecting point for uKIM-1. We stratified the included studies based on test times, 24 hours (detected uKIM-1 24 hours after contrast exposure), and <24 hours group (detected uKIM-1 24 hours before contrast exposure) and noticed that the values of AUC in 24 hour group were higher than those of the under 24 hours group. One of these studies detected uKIM-1 at both 6 hours and 24 hours after contrast and reported an AUC of 0.74 (95% CI 0.49–0.91, *P*=0.0064) and 0.85 (95% CI 0.72–0.95, *p*0.002), respectively. Another study [18] also illustrated that it is better to detect KIM-1 at 24 hours after contrast exposure. However, a study by Han et al. [29] showed that uKIM-1 levels did not rise significantly in patients of contrast-induced nephropathy. These mixed findings might be explained by differences in the renal inclusion criteria, inconsistent timing of urinary collections, and variable measurement methods.

Most of the included studies did not include patients with CKD, except for the study conducted by Wang et al. [10]. The results of the sensitivity analysis show that exclusion of CKD increased the stability of sensitivity. This indicates that the accuracy of uKIM-1 for predicting CI-AKI differs between patients with and without CKD. AKI that happened on CKD patients is known to be severe and different to recover [30]. The pathophysiological changes of CKD, such as the activation of TGF-*β*, p53, and HIF pathways in kidney and CKD related chronic inflammation and vascular dysfunction, might contribute to the severe AKI progress [30]. On the contrary, some clinical studies found that KIM-1 did not provide robust prognostic information on the loss or renal function in CKD population [31, 32]. Since the sample size was small in Wang et al. [10] research, studies with a larger sample size are needed to clarify this problem.

Recently, similar meta-analysis studies were done on the predictive value of KIM-1 in predicting AKI in different populations. However, the diagnostic value of KIM-1 varied. In a meta-analysis included general population, KIM-1 was found to be a promising biomarker for AKI prediction with an AUC of 0.86 [11]. In another study which only enrolled children, KIM-1 (AUC = 0.69) is not recommended for AKI diagnosis [33]. Those conflicted conclusions show that the diagnostic power of KIM-1might not be consistent in people of different ages. Further, KIM-1 only showed modest discrimination in diagnosing AKI in cardiac surgery patients [26] and showed stronger diagnostic power in our studies with patients received cardiac catherization treatment, since contrast is the main cause of AKI after cardiac catherization [34], while ischemia contributes to AKI after cardiac surgery in most cases [35]. The difference in pathophysiological mechanisms in these two conditions might lead to the discrepancy in KIM-1 diagnostic value on AKI.

## 5. Limitations

Limitations of this meta-analysis include the small number of studies included. Also, it was hard to assess influence of detecting time, as most studies detected uKIM-1 at different times but only one study reported the complete data. A few studies tested more than one biomarker; however, the details for the combined diagnosis of uKIM-1 and other biomarkers were not reported. Moreover, patients with different renal function and age were not accounted for (or controlled for) in most of the study designs.

## 6. Conclusion

The findings from this meta-analysis demonstrate that uKIM-1 is a relatively good biomarker for prediction CI-AKI in patients who undergo PCI or CAG. The optimal detection time is 24 hours after contrast exposure, suggesting that uKIM-1 can predict CI-AKI at an earlier time than sCr. Clinical studies with large sample sizes are needed to further explore the diagnostic ability and prognostic value of uKIM-1 in different populations (e.g., different age, renal function, and comorbidities).

## Figures and Tables

**Table 1:** Characteristics of included studies.

Author, country, year	Sample size	Study design	CI-AKI patients number	Inclusion criteria	Age, years N/C	Male N/C	SCr baseline N/C mmol/L	CI-AKI criteria	Hypertension % N/C	DM% N/C	CM type
Wybraniec et al., Poland, 2017 [22]	95	Prospective	9	Normal sCr	64 (52–75)	52 (54.4%)	70.72 (60.99–82.21)	≥50% relative or ≥0.3 mg/dL absolute increase of serum creatinine concentration at 48 h postprocedurally	48 (50.4%)	17 (17.6%)	Iopromide (LOCM) or Iodixanol (IOCM) or Iomeprol (LOCM)
Akdeniz et al., Turkey, 2015 [8]	52	Nested case-control	32	Normal sCr	67.5 ± 10.1/61.6 ± 9.4	11(34.4)/12(60.0)	79.56(44.2–167.96)	An increase in sCr by 0.3 mg/dL from a baseline level according to the KDIGO 2012 AKI criteria	-/0.5	81.3/85	Iodixanol
Li et al., China, 2015 [9]	145	Prospective	19	eGFR > 15 mL/min/1.73 m^2^	66.8 ± 9.9	91(62.8)	70.58 ± 9.9/60.41 ± 14.20	An increase of ≥0.5 mg/dl or ≥25% in SCr levels over the baseline 24–48 h after the intravascular injection of contrast medium, without an alternative etiology	NR	NR	Nonionic, low osmolarity contrast agent
Luo et al., China, 2013 [18]	42	Nested case-control	12	sCr < 1.5 mg/dL in male/sCr < 1.2 mg/L in female	68.4 ± 10.1/64.7 ± 13.2	5(41.7)/12(40.0)	68.76 ± 10.92/66.57 ± 12.89	An absolute rise in sCr of ≥0.5 mg/dl (44.2 mmol/l), and/or *a* ≥25% increase from the baseline within 48 hours	58.3/56.7	NR	Nonionic, low osmolarity contrast medium (iopamidol)
Torregrosa et al., Spain, 2015 [19]	144	Prospective	20	eGFR > 15 mL/min/1.73 m^2^	72 ± 10/62 ± 13	16(75)/94(75.8)	78.68 ± 21.22/100.78 ± 23.87	An increase in sCr ≥ 50 % according to the RIFLE classification system	NR	NR	NR
Vijayasimha et al., India, 2014 [17]	100	Prospective	12	sCr < 1.5 mg/dL in male/sCr < 1.2 mg/Ll in female	48.6 ± 11	62(62)	106.09 ± 15.03	An increase in sCr >25% of the baseline level 48 hours after contrast	NR	NR	Low osmolarity contrast (iodizanol or iopromide) medium.
Wang et al., China, 2014 [10]	42	Prospective	14	eGFR >15 mL/min/1.73 m^2^	60.2 ± 9.5/60.6 ± 8.1	7(50)/16(57)	82.2 ± 18.3/92.0 ± 15.5	An increase of ≥0.5 mg/dl or ≥25% in SCr levels over the baseline 24–48 h after the intra- vascular injection of contrast medium, without an alternative etiology	64/60	14/17	A nonionic, low osmolarity contrast agent 844 mOsm/kg
Wang, China, 2013 [21]	260	Prospective	20	sCr < 300 mmol/L	73.6 ± 8.9/60 ± 7.5	8(40)/135(56.3)	71.31 ± 8.3/65.7 ± 10.3	An increase of ≥0.5 mg/dl or ≥25% in SCr levels over the baseline 24–48 h after the intravascular injection of contrast medium, without an alternative etiology	50/20	15/5	a nonionic, low osmolarity contrast agent 600 mOsm/kg
Wang, China, 2014 [20]	66	Prospective	6	eGFR < 60 mL/min/1.73 m^2^	NR	NR	NR	An increase of ≥0.5 mg/dl or ≥25% in SCr levels over the baseline 24–48 h after the intravascular injection of contrast medium, without an alternative etiology	NR	NR	NR

**Table 2:** Performance of KIM-1 for CIN or CI-AKI diagnosis in the meta-analysis.

Nr.	Author	TP	FP	FN	TN	Total	Test time, hours	AUC	95% CI	SEN, %	SPE, %	Cutoff, ng/ml	Biological material	Assay
1	Wybraniec et al. [22]	7	15	2	71	95	6h	0.0081	NR	0.778	0.824	>0.425	Urine	ELISA
2	Akdeniz et al. [8]	24	5	8	15	52	6	0.797	0.677–0.917	75	75	3.66	Urine	ELISA
3	Li et al. [9]	14	18	5	108	169	24	0.854	0.782–0.929	73.7	85.7	6.327755	Urine	ELISA
4	Luo et al. [18]	10	4	2	26	42	24	0.85	0.72–0.95	83	87	0.74	Urine	ELISA
5	Torregrosa et al. [19]	14	45	6	79	144	12	0.713	0.551–0.876	71.6	64	1.73	Urine	ELISA
6	Wang and Pu [20]	12	8	2	20	42	24	0.839	0.095–0.948	85.7	71.4	4.495	Urine	ELISA
7	Wang [21]	17	60	3	180	260	12	0.903	0.802–0.100	85	75	0.0478425	Urine	ELISA
8	Wang et al. [10]	59	1	3	3	66	12	0.742	NR	95.2	75	3^*∗*^KIM-1 baseline	Urine	ELISA
9	Vijayasimha et al. [17]	11	17	1	71	100	24	0.95	NR	89	81	4.5	Urine	ELISA

**Table 3:** Subgroup analysis.

Subgroup	*N*	Sensitivity	Specificity	DOR	AUC
*Test time < 24 h*					
<24 h	5	0.84(0.70, 0.93)	0.72 (0.64, 0.79)	15 (5, 42)	0.81 (0.77–0.84)
24 h	4	0.83(0.70, 0.91)	0.83 (0.77, 0.87)	23 (11, 49)	0.89 (0.86–0.91)
ckd					
No	8	0.79 (0.71,0.86)	0.77 (0.70,0.83)	13 (7,24)	0.83 (0.80–0.86)
Yes	1	95.2	75		0.742

*Large sample size*					
≤100	4	0.79 (0.68,0.87)	0.77 (0.68,0.84)	13 (6,29)	0.84 (0.80–0.87)
>100	5	0.87 (0.75,0.94)	0.78 (0.67,0.86)	24 (9,69)	0.80 (0.76–0.83)

## Data Availability

The figures and tables data used to support the findings of this study are included within the article.

## Supplementary Materials

Supplementary Materials Figure S1: proportion of studies with low, high, or unclear concerns regarding applicability and risks of bias. Table S1: methodological quality assessment with QUADAS-2 tool for the 8 studies included in this meta-analysis. Checklist S1 PRISMA checklist: Preferred Reporting Items for Systematic Reviews and Meta-Analyses.

## References

[1] Andreucci M., Faga T., Riccio E., Sabbatini M., Pisani A., Michael A. (2016). The potential use of biomarkers in predicting contrast-induced acute kidney injury. *International Journal of Nephrology and Renovascular Disease*.

[2] Sudarsky D., Nikolsky E. (2011). Contrast-induced nephropathy in interventional cardiology. *International Journal of Nephrology and Renovascular Disease*.

[3] Bragadottir G., Redfors B., Ricksten S.-E. (2013). Assessing glomerular filtration rate (GFR) in critically ill patients with acute kidney injury-true GFR versus urinary creatinine clearance and estimating equations. *Critical Care*.

[4] Stacul F., van der Molen A. J., Reimer P. (2011). Contrast induced nephropathy: updated ESUR contrast media safety committee guidelines. *European Radiology*.

[5] Ichimura T., Asseldonk E. J. P. V., Humphreys B. D., Gunaratnam L., Duffield J. S., Bonventre J. V. (2008). Kidney injury molecule-1 is a phosphatidylserine receptor that confers a phagocytic phenotype on epithelial cells. *Journal of Clinical Investigation*.

[6] Bonventre J. V. (2009). Kidney injury molecule-1 (KIM-1): a urinary biomarker and much more. *Nephrology Dialysis Transplantation*.

[7] Ichimura T., Bonventre J. V., Bailly V. (1998). Kidney injury molecule-1 (KIM-1), a putative epithelial cell adhesion molecule containing a novel immunoglobulin domain, is up-regulated in renal cells after injury. *Journal of Biological Chemistry*.

[8] Akdeniz D., Celik H. T., Kazanci F. (2015). Is kidney injury molecule 1 a valuable tool for the early diagnosis of contrast-induced nephropathy?. *Journal of Investigative Medicine: The Official Publication of the American Federation for Clinical Research*.

[9] Li W., Yu Y., He H., Chen J., Zhang D. (2015). Urinary kidney injury molecule-1 as an early indicator to predict contrast-induced acute kidney injury in patients with diabetes mellitus undergoing percutaneous coronary intervention. *Biomedical Reports*.

[10] Wang F., Zhao Q., Peng C. (2014). Significance of combined detection of cys-C, ngal and kim-1 in contrast induced nephropathy after coronary angiography. *European Journal of Experimal Biology*.

[11] Xinghua Shao L. T., Xu W., Zhang Z. (2014). Diagnostic value of urinary kidney injury molecule 1 for acute kidney injury: a meta-analysis. *PLoS One*.

[12] Liberati A., Altman D. G., Tetzlaff J. (2009). The PRISMA statement for reporting systematic reviews and meta-analyses of studies that evaluate health care interventions: explanation and elaboration. *Journal of Clinical Epidemiology*.

[13] B. A. Dwamena, RSARCC. midas: meta-analysis of diagnostic accuracy studies

[14] van Houwelingen H. C., Arends L. R., Stijnen T. (2002). Advanced methods in meta-analysis: multivariate approach and meta-regression. *Statistics in Medicine*.

[15] Higgins J. P. T., Thompson S. G. (2002). Quantifying heterogeneity in a meta-analysis. *Statistics in Medicine*.

[16] Chappell F. M., Raab G. M., Wardlaw J. M. (2009). When are summary ROC curves appropriate for diagnostic meta-analyses?. *Statistics in Medicine*.

[17] Vijayasimha M., Padma V., Mujumdar S. K., Satyanarayana P. V. V., Yadav A. (2014). Kidney injury molecule-1: a urinary biomarker for contrast-induced acute kidney injury. *Medical Journal of Dr. D.Y. Patil University*.

[18] Luo Q., Zhou F., Dong H. (2013). Implication of combined urinary biomarkers in early diagnosis of acute kidney injury following percutaneous coronary intervention. *Clinical Nephrology*.

[19] Torregrosa I., Montoliu C., Urios A. (2015). Urinary KIM-1, NGAL and L-FABP for the diagnosis of AKI in patients with acute coronary syndrome or heart failure undergoing coronary angiography. *Heart and Vessels*.

[20] Wang L., Pu X. (2014). Predict value of monitoring changes of urinary neutrophil gelatinase-associated lipocalin and kidney injury molecule-1 after coronary angiography and percutaneous coronary intervention on early diagnosis of contrast-induced nephropathy. *Chinese Journal of Cardiology*.

[21] Wang D. (2013). Assessment of urinary cystatin C and KIM-1 on contrast induced acute kidney injury after coronary andiograph.

[22] Wybraniec M. T., Chudek J., Bożentowicz-Wikarek M., Mizia-Stec K. (2017). Prediction of contrast-induced acute kidney injury by early post-procedural analysis of urinary biomarkers and intra-renal Doppler flow indices in patients undergoing coronary angiography. *Journal of Interventional Cardiology*.

[23] Nickolas T. L., Schmidt-Ott K. M., Canetta P. (2012). Diagnostic and prognostic stratification in the emergency department using urinary biomarkers of nephron damage. *Journal of the American College of Cardiology*.

[24] Han W. K., Wagener G., Zhu Y., Wang S., Lee H. T. (2009). Urinary biomarkers in the early detection of acute kidney injury after cardiac surgery. *Clinical Journal of the American Society of Nephrology*.

[25] Vaidya V. S., Waikar S. S., Ferguson M. A. (2008). Urinary biomarkers for sensitive and specific detection of acute kidney injury in humans. *Clinical and Translational Science*.

[26] Ho J., Tangri N., Komenda P. (2015). Urinary, plasma, and serum biomarkers’ utility for predicting acute kidney injury associated with cardiac surgery in adults: a meta-analysis. *American Journal of Kidney Diseases*.

[27] Wang K., Duan C.-Y, Wu J. (2016). Predictive value of neutrophil gelatinase-associated lipocalin for contrast-induced acute kidney injury after cardiac catheterization: a meta-analysis. *Canadian Journal of Cardiology*.

[28] Sabbisetti V. S., Waikar S. S., Antoine D. J. (2014). Blood kidney injury molecule-1 is a biomarker of acute and chronic kidney injury and predicts progression to ESRD in type I diabetes. *Journal of the American Society of Nephrology*.

[29] Han W. K., Bailly V., Abichandani R., Thadhani R., Bonventre J. V. (2002). Kidney Injury Molecule-1 (KIM-1): a novel biomarker for human renal proximal tubule injury. *Kidney International*.

[30] He L., Wei Q., Liu J. (2017). AKI on CKD: heightened injury, suppressed repair, and the underlying mechanisms. *Kidney International*.

[31] Bhavsar N. A., Köttgen A., Coresh J., Astor B. C. (2012). Neutrophil gelatinase-associated lipocalin (NGAL) and kidney injury molecule 1 (KIM-1) as predictors of incident CKD stage 3: the atherosclerosis risk in communities (ARIC) study. *American Journal of Kidney Diseases*.

[32] Black L. M., Lever J. M., Traylor A. M. (2018). Divergent effects of AKI to CKD models on inflammation and fibrosis. *American Journal of Physiology-Renal Physiology*.

[33] Fazel M., Sarveazad A., Mohamed Ali K., Yousefifard M., Hosseini M. (2020). Accuracy of urine kidney injury molecule-1 in predicting acute kidney injury in children; a systematic review and meta-analysis. *Archives of Academic Emergency Medicine*.

[34] Fähling M., Seeliger E., Patzak A., Persson P. B. (2017). Understanding and preventing contrast-induced acute kidney injury. *Nature Reviews Nephrology*.

[35] Wang Y., Bellomo R. (2017). Cardiac surgery-associated acute kidney injury: risk factors, pathophysiology and treatment. *Nature Reviews Nephrology*.

