# Supplementary figures and images for: The Predictive Value of Urinary Kidney Injury Molecular 1 for the Diagnosis of Contrast-Induced Acute Kidney Injury after Cardiac Catheterization: A Meta-Analysis
Source: J Interv Cardiol. 2020 Aug 13;2020:4982987. doi: 10.1155/2020/4982987 (PMC7443242; doi:10.1155/2020/4982987)

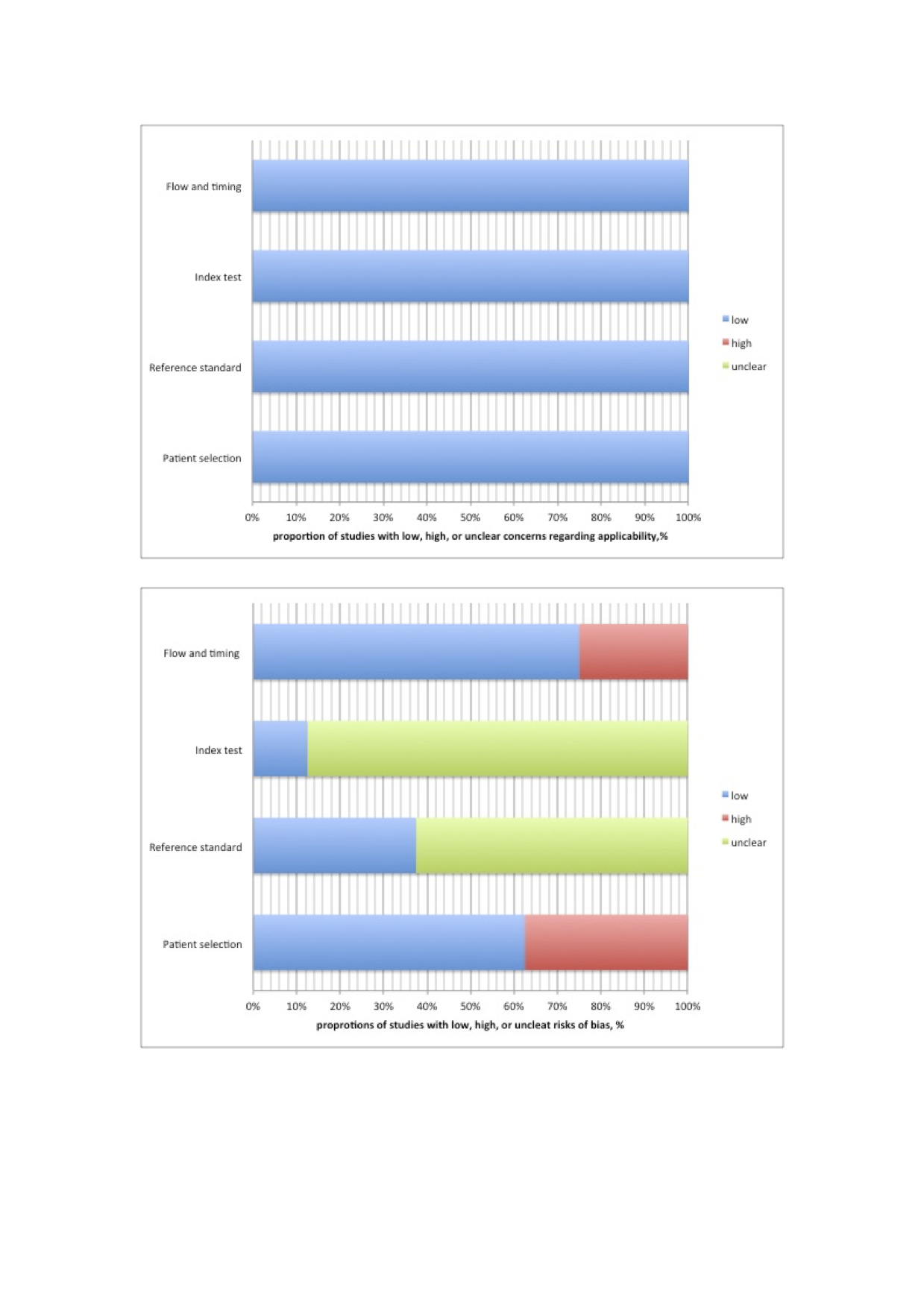

Supplement: Supplementary Materials — Figure S1: proportion of studies with low, high, or unclear concerns regarding applicability and risks of bias. Table S1: methodological quality assessment with QUADAS-2 tool for the 8 studies included in this meta-analysis. Checklist S1 PRISMA checklist: Preferred Reporting Items for Systematic Reviews and Meta-Analyses. [file 4982987.f1.zip › 4982987.f1/Figure S1.tiff]
